# Gamma smooth muscle actin as a new potential marker of cancer-associated fibroblasts

**DOI:** 10.1007/s00418-025-02419-9

**Published:** 2025-09-19

**Authors:** Michal Španko, Lucie Pfeiferová, Eliška Drobná Krejčí, Michal Kolář, Pavel Dundr, Jaroslav Valach, Karel Smetana, Lukáš Lacina

**Affiliations:** 1 Institute of Anatomy, First Faculty of Medicine, Charles University, Prague, Czech Republic; 2 Department of Stomatology, First Faculty of Medicine, Charles University, Prague, Czech Republic; 3 Institute of Molecular Genetics, Czech Academy of Sciences, Prague, Czech Republic; 4 Institute of Pathology, First Faculty of Medicine, Charles University, Prague, Czech Republic; 5 General University Hospital, Prague, Czech Republic

**Keywords:** Cancer-associated fibroblast, γ-smooth muscle actin, Epithelial-mesenchymal transition, *TPD52L1*, *CLEC12A*

## Abstract

Cancer-associated fibroblasts (CAFs) represent an important component of the cancer ecosystem, influencing the broad scale of biological properties of tumour cells, including the capacity for metastasis formation. An important CAF subpopulation, known as myCAFs, typically expresses α-smooth muscle actin. Transcriptomic analysis demonstrated that activated fibroblasts isolated from various pathological tissues also express the *ACTG2* gene encoding γ-smooth muscle actin. This was further validated by immunocytochemistry. Using the scratch test in vitro*,* it was possible to demonstrate that γ-smooth muscle actin may be associated with the epithelial-mesenchymal transition, which was also shown by transcriptomic analysis. The presence of γ-smooth muscle actin-positive fibroblasts in histopathological sections of human tumours verified the expression of this protein as a new potential marker of CAFs.

**Supplementary Information:**

The online version contains supplementary material available at 10.1007/s00418-025-02419-9.

## Introduction

Tumours contain a mix of different cell types besides cancer cells. These stromal components are represented by non-malignant cell types, such as various immune cells, endothelial cells of blood and lymphatic vessels, pericytes, and cancer-associated fibroblasts (CAFs). Such a diverse assembly of cell types of malignant and non-malignant nature form a highly orchestrated complex ecosystem, exerting a multitude of functions (Swanton et al. 2024).

In this context, CAFs generally support the maintenance of low differentiation status in cancer cells (Clevers 2011). CAFs also affect cancer cell proliferation and modulate their migration capacity through the tissue and potential metastatic spread via angiogenesis (Lacina et al. 2023). Activated CAFs can promote extracellular matrix remodelling and thus even contribute to chemoresistance (Lin et al. 2024) and radioresistance (Zhang et al. 2023). On the other hand, CAFs can also trigger fibroplasia, which is believed to be a tumour-suppressing mechanism in some tumours, namely those of the pancreas. Such diversity of functions is likely to reflect the heterogeneity of CAFs. Indeed, extensive research in previous years demonstrated that CAFs represent a heterogeneous group of fibroblasts with various transcriptional programs (Novotný et al. 2020; Chen et al. 2024; Cords et al. 2024). The protein production by fibroblasts of the tumour stroma vastly depends on their differentiation status and also on their origin (Liu et al. 2024).

This knowledge can be therapeutically relevant because some of the CAF-produced proteins can significantly regulate the biology of cancer cells. Thus, CAFs represent a potential target for the therapeutic manipulation of pathways controlling the interaction of cancer cells with their microenvironment (Chen et al. 2024).

Actin family members are key structural proteins that build the cytoskeleton of cells. Actins participate in cell division, migration, and vesicle trafficking. This protein family comprises six different cell-type-specific isoforms: ACTA1, ACTA2, ACTB, ACTC1, ACTG1, and ACTG2. Actin isoforms have been extensively studied in many cancers (Suresh and Diaz 2021).

For diagnostic purposes, αSMA is traditionally considered a valuable histochemical marker for CAFs. However, this does not label every individual fibroblast-like cell of the tumour stroma. Many αSMA-negative cells with otherwise fibroblast-like morphology are present across the cancer niche (Lacina et al. 2023). Moreover, the αSMA-rich phenotype is consistent with myofibroblasts occurring in the granulation tissue during wound healing (Gál et al. 2022). Thus, this highly convenient marker identifies merely a CAF subpopulation of the tumour stroma. Because of their similarity to myofibroblasts, we and others refer to them as myCAFs (Zeltz et al. 2019; Monteran and Erez 2023). Unfortunately, no universal, highly specific single marker of CAFs has been discovered to date (Han et al. 2020).

Despite αSMA having undisputed precedence in tumour biology due to the wide range of molecular and cellular interactions in which it is involved, it is not the only actin family member present in the tumour microenvironment. Gamma-smooth muscle actin (γSMA) is also referred to as the “enteric form”. This form was initially studied because its mutations are associated with serious visceral myopathy of the digestive tube (Collins et al. 2019).

However, the γSMA presence is not exclusive to the intestine. The expression of γSMA in dermal fibroblasts seems to coincide with the phase of scar remodelling (Bond et al. 2011). It was also observed in CAFs in head and neck squamous cell carcinomas (Strnad et al. 2010). Further, γSMA was also observed in the process of the epithelial-to-mesenchymal transition of cells of hepatocellular carcinoma (Benzoubir et al. 2015).

In this study, we aimed to address the similarities and differences of αSMA and γSMA, two actin family members, in the tumour stroma of various cancer types. First, we aimed to compare the immunohistochemical detection of αSMA and γSMA in tissue sections using a broad collection of human tumours. To observe the dynamics of SMA expression, we performed immunocytochemical detection of γSMA in vitro in cancer cells using the scratch test. To visualize co-expression, we also used double labelling of both studied types of SMA in these experiments.

To elucidate the consequences of γSMA expression in cells of the cancer ecosystem, CAFs from malignant cutaneous melanoma (MELF), ocular melanoma (oMELF), squamous cell carcinoma of the head and neck (SCCF), and ductal adenocarcinoma of the pancreas (PANF) were analysed. We used primary dermal fibroblasts (DF) isolated from normal human skin as a control. Further, we also tested normal fibroblasts from the pancreas affected by cancer (PANF_control) and fibroblasts prepared from the skin of a patient with systemic scleroderma (SSF). It is widely known that in both these non-malignant conditions, activated fibroblasts are also present.

## Materials and methods

### Cell culture for transcriptomic analysis and in vitro experiments

a. Fibroblasts

Fibroblasts were isolated using a protocol published earlier (Dvořánková et al. 2019) from samples of normal skin, skin from adult patients who have systemic scleroderma, and various tumours (summarized in Table 1). All tissue samples were acquired with the approval of the local ethics committees of the General University Hospital, University Hospital Královske Vinohrady, and University Hospital Motol (both in Prague, Czech Republic) with the explicit written informed consent of all individuals. Cells exhibiting positivity for vimentin and negativity for keratins, CD34, CD45, and MELAN-A or HMB45 were considered fibroblasts (Dvořánková et al. 2019). The cells were cultured under standard conditions at 37 °C and 5% CO_2_ atmosphere in Dulbecco’s modified Eagle’s medium (DMEM) containing 10% foetal bovine serum, as described elsewhere (Strnadová et al. 2022). We used cells before reaching the fifth passage for all in vitro experiments, including transcriptomic analysis.

**Table 1:** Collection of isolated fibroblasts

Type of fibroblasts	Abbreviation	No. of samples
Normal dermal fibroblasts	DF	3
Fibroblasts (dermal) from systemic sclerosis	SSF	2
Fibroblasts from the pancreas—collected distant from ductal adenocarcinoma tissue	PANF_control	1
CAFS from squamous cell carcinoma	SCCF	2
CAFs from ocular malignant melanoma	oMELF	1
CAFs from cutaneous malignant melanoma	MELF	4
CAFs from ductal adenocarcinoma of the pancreas	PANF	7

b. FaDu cells and scratch test

A commercially available cell line of the squamous cell carcinoma of the hypopharynx was obtained from ATCC (Manassas, VA, USA), cultured as described above (FaDu, CVCL_1218). These cells were used for in vitro scratch tests.

FaDu cells were seeded at a density of 50,000/cm^2^ on sterilized, pre-cleaned coverslips and cultured to confluence under the above-described conditions. The scratch test was performed 48 h after the monolayer reached full confluence using a well-established protocol (Liang et al. 2007). After 24 h, coverslips were washed with phosphate-buffered saline (PBS) and fixed in 4% paraformaldehyde at room temperature.

### Immunocytochemistry and immunohistochemistry

For immunocytochemical detection of αSMA, we used a widely recognized mouse monoclonal antibody (clone 1A4) suitable for immunohistochemistry, immunofluorescence, flow cytometry, and western blotting produced by Abcam (Cambridge, UK). For immunization, the N-terminal decapeptide of the αSMA isoform of actin was selected, providing isoform specificity. Further, we used a commercial rabbit polyclonal antibody raised against the N-terminus of γSMA (a synthetic peptide representing human ACTG2 amino acid residues 1–50 was used for immunization; ab189385 produced by Abcam).

FITC-labelled phalloidin diluted as recommended by the supplier (Thermo Fisher Scientific, Waltham, MA, USA) was employed for the detection of F-actin.

For immunohistochemical analysis, formalin-fixed paraffin-embedded tissue blocks were obtained from the archive of the Institute of Pathology (First Faculty of Medicine, Charles University and General University Hospital, Prague). The collection of human tumours used in this study is summarized in Table 2. Consecutive sections (5 μm thickness) were prepared from each type of tumour (Table 1). Sections were routinely processed, including deparaffination and antigen retrieval (pH 6.0, 120 °C in citrate buffer), followed by hydrogen peroxide blocking (1% in PBS, 30 min, room temperature). Non-specific reactivity was blocked by 10% goat serum (30 min, room temperature).

**Table 2:** Collection of studied tumours and the number of samples positive for αSMA/γSMA

Type of tumour	Number of samples	Number of positive samples (αSMA/γSMA)
High-grade serous carcinoma of the ovary	8	4/3
Mucinous adenocarcinoma of the ovary	2	0/0
Ductal adenocarcinoma of the pancreas	6	3/3
Metastasis of colon adenocarcinoma to the ovary	6	4/4
Adenocarcinoma of the rectum	6	3/3
Metastasis of appendix adenocarcinoma to the ovary	4	4/4
Squamous cell carcinoma of the oral cavity, including the tongue	6	4/5
Squamous cell carcinoma of the skin	5	3/3
Squamous cell carcinoma of the uterine cervix	6	4/4
Squamous cell carcinoma of the oesophagus	6	5/5
Cutaneous melanoma	4	0/0

Antibodies against αSMA and γSMA (as above) were diluted 1/100 and incubated overnight at 4 °C according to supplier recommendations. Isotype controls verified the specificity of immunodetection for each primary antibody (Thermo Fisher Scientific, Waltham, MA, USA). The secondary antibodies for immunohistochemical detection were polymer HRP-labelled (Histofine® Simple Stain MAX PO (MULTI), Nichirei Biosciences, Tokyo, Japan). For immunofluorescence, we used goat anti-mouse IgG and goat anti-rabbit IgG antibodies (Invitrogen/Thermo Fisher Scientific) conjugated with Alexa 488 and Alexa 555 fluorochromes, respectively. Nuclei were counterstained by Gill’s haematoxylin or by DAPI for immunofluorescence.

### Microscopy

Imaging was performed on a Leica Thunder Imager system, AF 6000LX, microscope type DM6B-Z (Leica Microsystems CMS GmbH, Wetzlar, Germany), equipped with a camera (Leica-DFC9000GT-VSC10032), using software LAS X 3.9.0.28093 HardwareServerVersion = Build 28093. Individual nosepieces are as follows: 10x/0.32 DRY HC PL Fluotar, 20x/0.80 DRY HC PL APO, 40x/0.95 DRY HC PL APO. Physical setting: 2048 × 2048, Resolution = 16 (bit depth).

Fluorochromes used were as follows: Alexa 488, Alexa 555, DAPI. Filters used were as follows: DAP EX: 350/50, DC:400, EM:460/50; GFP EX:470/40, DC: 495, EM: 525/50; RHO EX: 546/10, DC: 560, EM:585/40. Mounting Medium Refraction Index = 1.4.

Pixel size = 0.0000065; Gamma = 1; Brightness = 50; WhiteValue = 65,535; BlackValue = 0; Binning = No Binning

### Transcriptome profiling on the array

Fibroblast populations were seeded at a density of 1000 cells/cm^2^ into two 6-cm Petri dishes (Corning, NY, USA) and cultured for 7 days (reaching 95–100% confluence). The culture medium was refreshed every 2 days and 24 h before cell harvest. Following that, the cells were washed twice with PBS (Biochrom), and 350 μl of RLT buffer (Qiagen, Hilden, Germany) with 2-mercaptoethanol (Sigma-Aldrich) was added. The resulting cell lysates were collected and immediately stored at –80 °C.

Total RNA was extracted using the RNeasy Micro Kit (Qiagen) following the standard procedure for animal cells. RNA quantity and quality were measured using an Agilent 2100 Bioanalyzer (Agilent Technologies, Santa Clara, CA, USA), ensuring that all samples had an RNA integrity number above 9. A total of 200 ng of RNA was amplified using the Illumina TotalPrep RNA Amplification Kit (Ambion; Thermo Fisher Scientific, Waltham, MA, USA), and 750 ng of the amplified RNA was hybridized onto Illumina HumanHT-12 v4 chips (Illumina, San Diego, CA, USA) according to the manufacturer’s protocol.

Raw data pre-processing was conducted using GenomeStudio software (version 1.9.0.24624; Illumina) and further analysed with the oligo (Carvalho and Irizzarry 2010) and limma (Ritchie et al., 2015) packages from Bioconductor (Huber et al. 2015), following the methodology outlined in Novak et al. (2021). Transcription profiles were background-corrected using a normal-exponential model, quantile-normalized, and variance-stabilized through base-2 logarithmic transformation. A moderated *t*-test was applied to identify differentially expressed transcripts (after fitting a linear model I ~ group + sex).

For visualization purposes in heatmaps and boxplots, all technical replicates within each biological sample were merged and averaged. Additionally, probe signals corresponding to the same gene were aggregated by computing their average expression values. In total, we analysed 21 biological samples.

## Results

### Immunohistochemical detection of αSMA and γSMA in paraffin sections from human cancer tissues

αSMA- and γSMA-positive CAFs were observed in tissue sections from routine clinical samples of a broad panel of human malignant tumours (Table 1).

To verify the specificity of antibodies against αSMA or γSMA, we detected both types of SMA in sections of normal skin and muscle (Supplementary Figure 1). Positive signals for both studied types of SMA were detected in smooth muscle cells of the vascular wall and of arrector pili muscle as well as myoepithelial cells of the sweat glands. Striated muscle fibres were positive for γSMA, and not for αSMA (Supplementary Figures 1 and 2).

In our sample collection, particular tumour types and individual samples within one category varied markedly in the extent of the stromal component and in the number of immunostained CAFs (Kellermann et al. 2008). Some tumour types (e.g., melanoma and mucinous adenocarcinoma of the ovary) revealed no or only negligible positivity (CAFs were not stained with αSMA, or less than 1% of stromal fibroblasts were stained with αSMA). Staining of smooth muscle cells of the blood vessels with αSMA was used as an internal positive control. In samples counted as positive, we usually observed “scanty” findings—using the terminology coined earlier by Kellerman and co-workers (Kellermann et al. 2008). In such cases, more than 1% and less than 50% of myofibroblasts were stained with αSMA. In our collection, we did not observe abundant staining, with more than 50% of CAFs stained with αSMA.

**Fig. 1:**
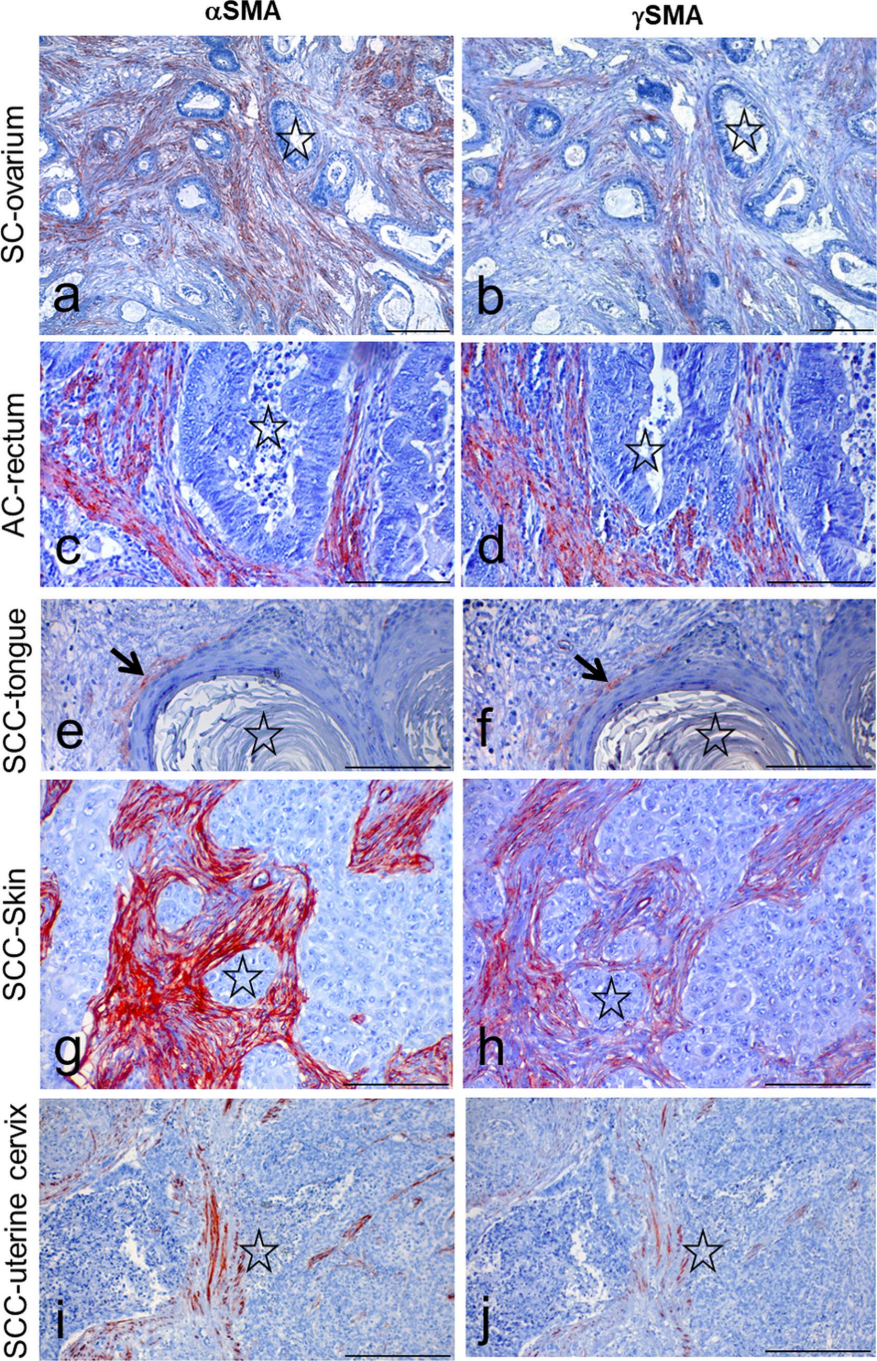
Detection of αSMA (**a**, **c**, **e**, **g**, **i**) and γSMA (**b**, **d**, **f**, **h**, **j**) in parallel sections from high-grade serous carcinoma of the ovary (SC-ovary, **a**, **b**), adenocarcinoma of the rectum (AC-rectum, **c, d**), squamous cell carcinoma of the tongue (SCC-tongue, e,f), squamous cell carcinoma of the skin (SCC-skin, **g**, **h**), and squamous cell carcinoma of the uterine cervix (SCC-uterine cervix, **i**, **j**). The figures demonstrate the matching position of αSMA- and γSMA-positive CAFs. Corresponding structures are marked by an asterisk. Arrows indicate a thin layer of cells positive for both types of SMA. Counterstained by Gill’s haematoxylin. The bar is 100 μm

Further, we also detected a difference in the immunostained CAF distribution pattern within the stroma. The representative positive areas are compared in Fig. 1.

In positive samples, we observed all three typical patterns of myofibroblast distribution within the stroma (Seifi et al. 2010). In some cases, the stroma was more abundant with a so-called network pattern, where positive cells were arranged in multiple rows with the interwoven network in the tumour stroma (Fig. 1a, b, g, h). In many tumours, we observed focal distribution, where immunostained CAFs had a focal arrangement or had no special arrangement in different stromal areas (Fig. 1c, d, i, j). Lastly, we detected the spindle pattern, with positive cells arranged in one to three rows surrounding the periphery of the neoplastic tumour buds (Fig. 1e, f); this was usually attached to highly differentiated regions of squamous cell carcinomas. We did not observe any particular stromal SMA pattern that is exclusive to any cancer type studied in our collection. Also, we did not observe positive staining in areas of epithelial tissue across the studied tumour types listed in Table 2.

### Transcriptomic analysis and bioinformatics analysis

All types of CAFs (MELF, oMELF, PANF, SSCF) and pathologically activated fibroblasts (PANF_control and SSF) differed significantly from the normal primary dermal fibroblasts (DF). This is evident from the heatmap presenting the transcriptome across all studied cells (Supplementary Figure 3).

**Fig. 2:**
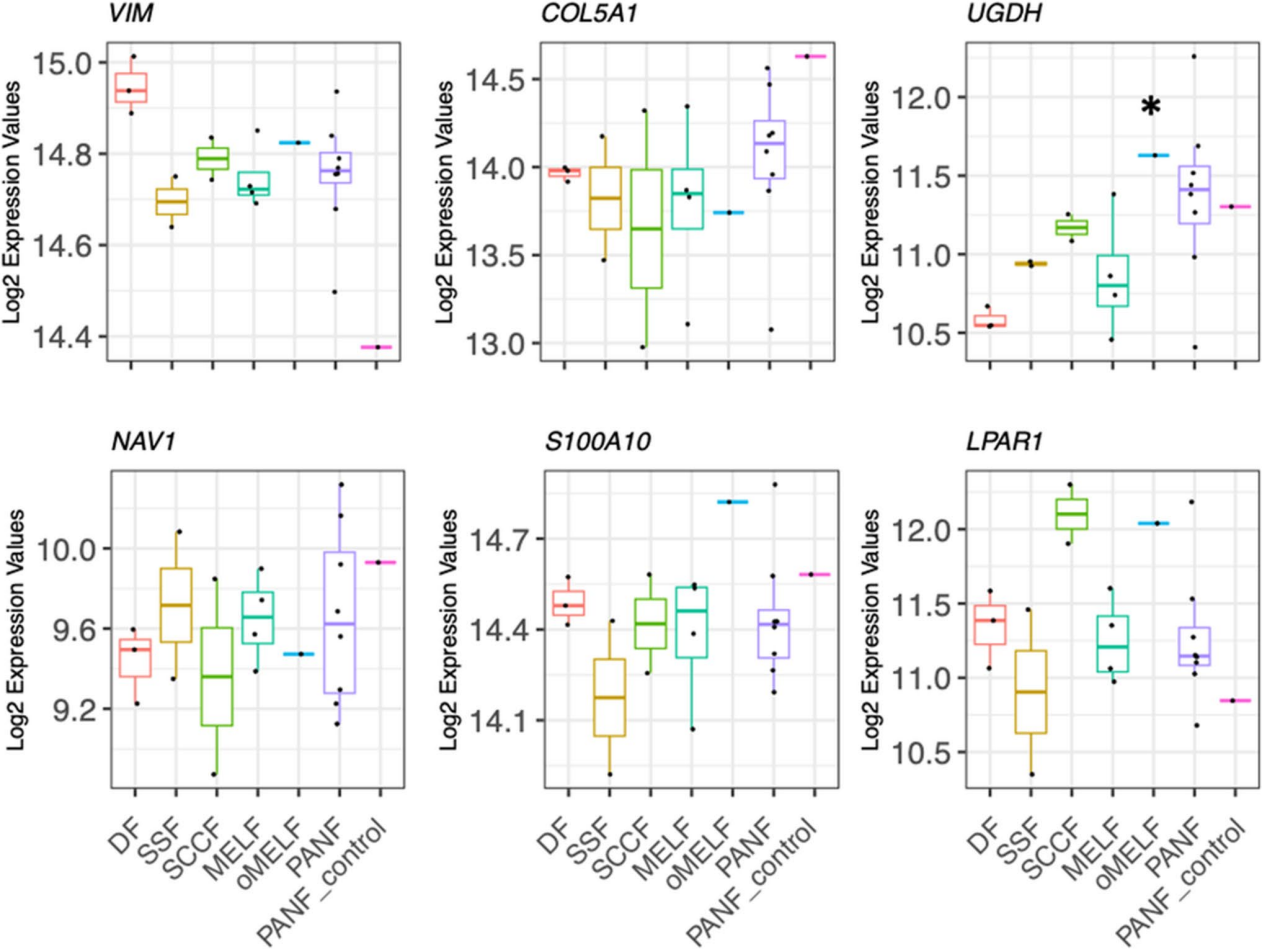
High expression of selected genes typical of fibroblasts. The asterisk indicates significantly differing expression from normal dermal fibroblasts (DF) at *p* < 0.05

To verify that all cells used in the study were indeed fibroblasts, we used a panel of genes proposed as criteria for fibroblast identification and discrimination, as described earlier on the basis of single-cell analysis (Muhl et al. 2020). This panel included *VIM*, *NAV1*, *COL5A1*, *S100A10*, *LPAR1,* and *UGDH*—all genes typical of their affiliation to this quite heterogeneous cell group. We confirmed that all these genes were active in the studied cells (Fig. 2).

**Fig. 3:**
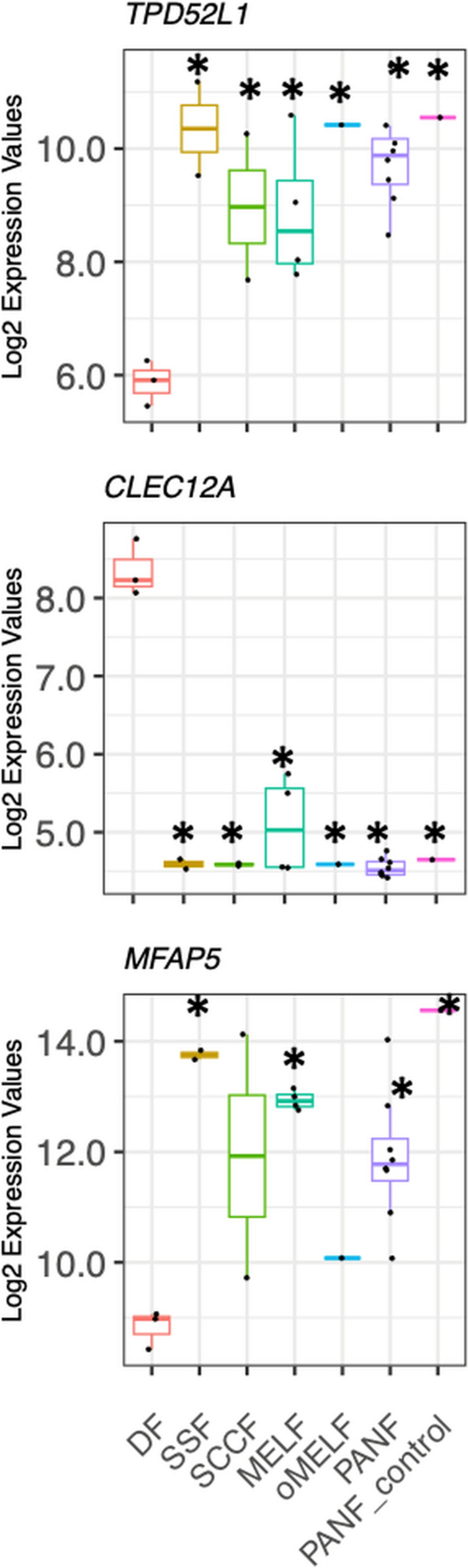
The activity of *TPD52L1*, *CLEC12A*, and *MFAP5* genes in fibroblasts from pathological tissue differs from that in normal dermal fibroblasts (DF). Arrows indicate a significant difference from DF (*p* < 0.05)

While addressing the principal difference between normal DF and fibroblasts prepared from all pathological tissues (all CAFs, and also PANF_control, SSF included), we discovered deficient activity of gene *TPD52L1* in normal DF, which contrasts with high activity of this gene in all fibroblasts from the abnormal tissues. Except for oMELF, all types of fibroblasts isolated from pathological tissues also exhibited activated gene *MFAP5* (Fig. 3). Conversely, we identified gene *CLEC12A as* active in normal DF, while its activity across all studied CAFs and also PANF_control and SSF was switched off (Fig. 3).

**Fig. 4:**
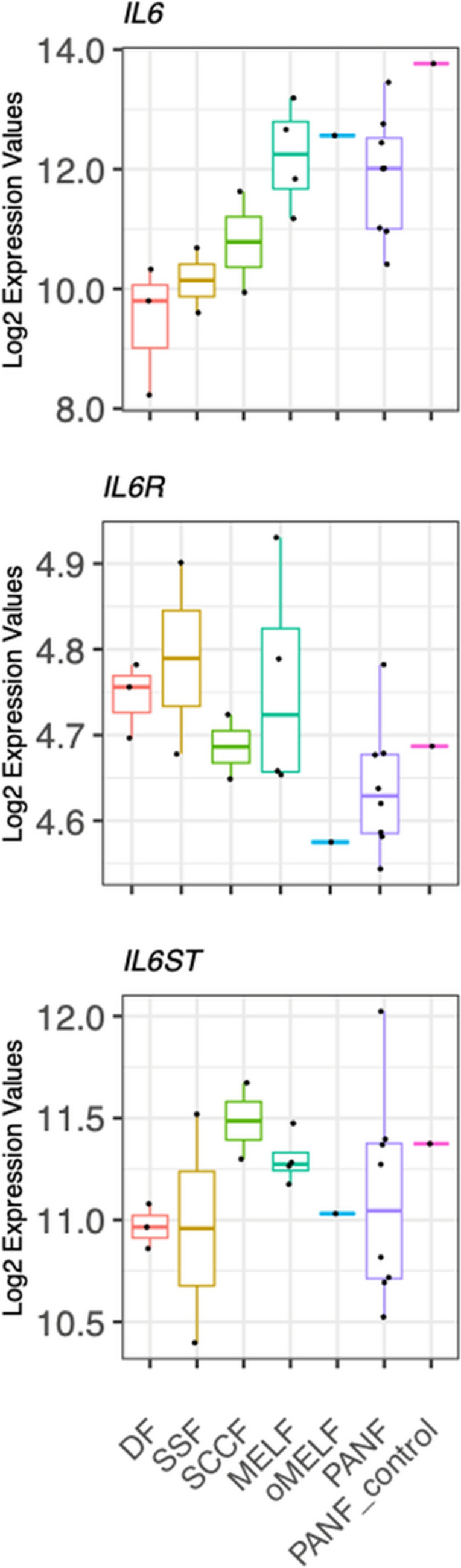
The activity of genes encoding IL-6 (*IL6*), IL-6 receptor (*IL6R*), and IL-6 signal transducer (*IL6ST*). The activity of *IL6* was insignificantly upregulated in fibroblasts originating from pathological tissue, contrary to normal dermal fibroblasts (DF). The activity of *IL6R* was minimal in all types of studied cells. In contrast, *IL6ST* was highly expressed in all fibroblasts. The differences between DF and other cell types were not statistically significant

CAFs are known as potent producers of IL-6 (Lacina et al. 2019), one of the important mediators of the intercellular dialogue between cancer and non-cancer cells (Geng et al. 2021; Rašková et al. 2022). Therefore, we investigated the activity of genes encoding IL-6 (*IL6*), the receptor for IL-6 (*IL6R*), and glycoprotein 130, the so-called signal transducer (*IL6ST*). The activity of the *IL6* gene was insignificantly higher in all fibroblasts from pathological tissue than that in normal DF. The activity of gene *IL6R* was low in all studied fibroblasts, despite active ongoing expression of *IL6ST* (Fig. 4).

**Fig. 5:**
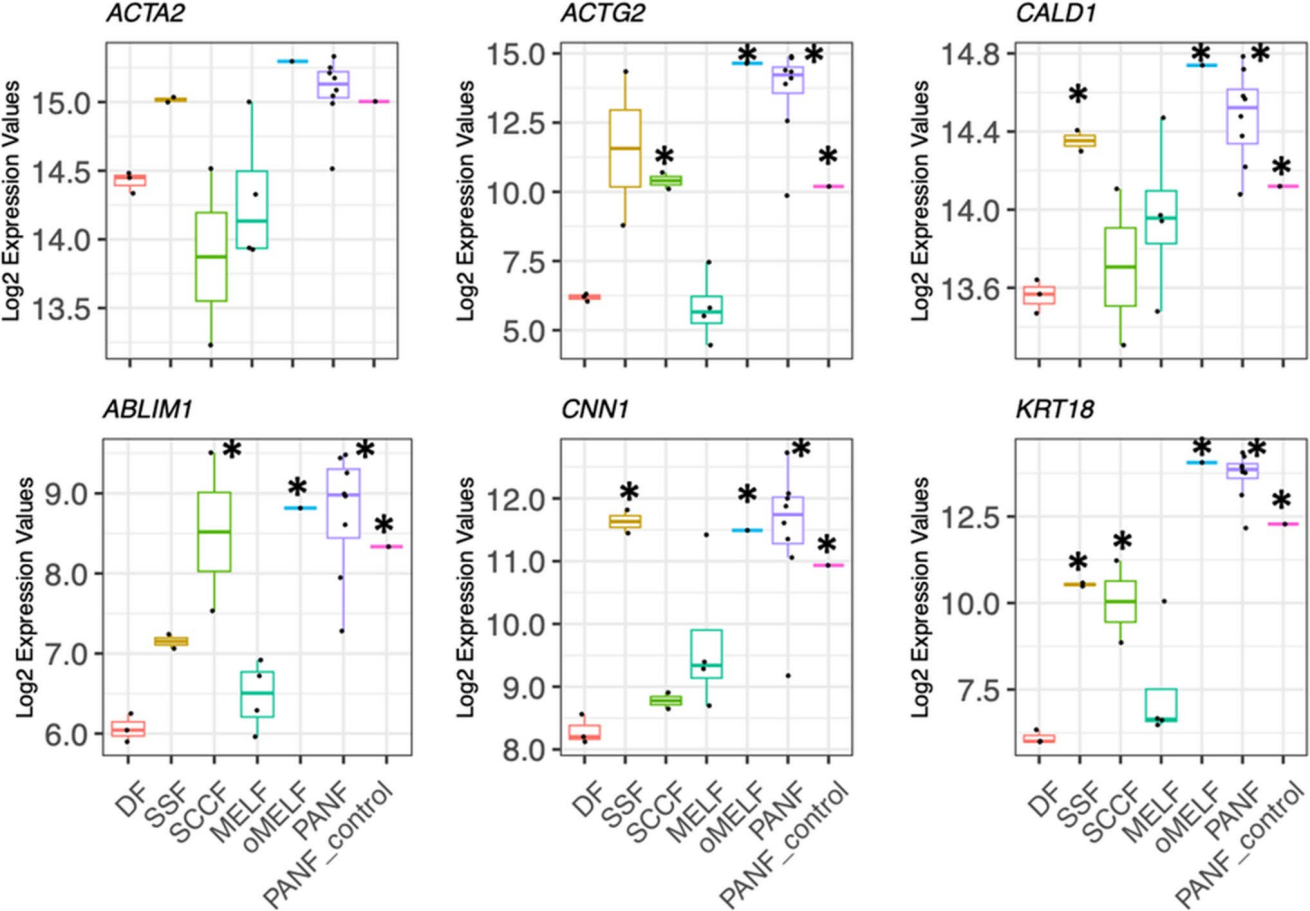
Fibroblasts exhibit active genes for αSMA (*ACTA2*), and except for MELF, exhibit activity of the gene (*ACTG2*) encoding γSMA. Interestingly, the activity of genes *ABLIM1* (protein responsible for interaction of actin with other proteins), *CNN1* (actin filament-associated regulatory protein), and *CALD1* (Ca^2^-dependent inhibition of smooth muscle contraction) was similar to *ACTG2*. Similar expression of the *KRT18* gene encoding keratin 18 is demonstrated. Significant differences from normal dermal fibroblasts (DF) (*p* < 0.05) are marked by an asterisk

High activity of gene *ACTA2* encoding αSMA was detected in all types of studied fibroblasts (Fig. 5). Surprisingly, we also detected high activity of the *ACTG2* gene encoding γSMA in PANF, PANF_control, SCCF, SSF, and oMELF in comparison with MELF and normal DF (Fig. 5). Very similar patterns of activity were also observed for *ABLIM1*, *CNN*, and *CALD1* genes except for SCCF cells (Fig. 5). Surprisingly, PANF, PANF_c, SCCF, SSF, and oMELF expressed the transcript for keratin 18 (*KRT18*) (Fig. 5).

### In vitro experiments and immunocytochemistry

**Fig. 6:**
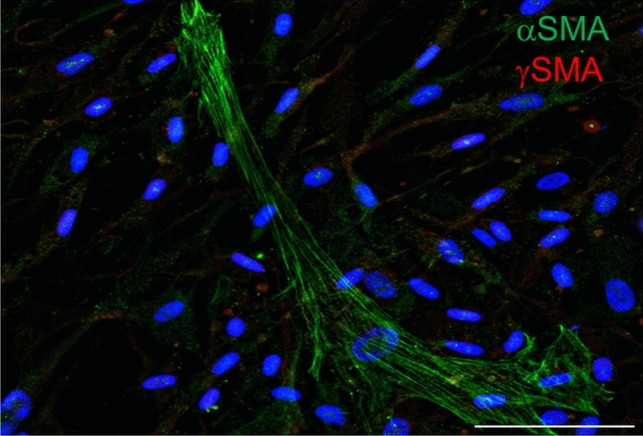
Detection of αSMA and γSMA in the culture of normal dermal fibroblasts. Normal dermal fibroblast cultures (DF) rarely contained αSMA-exhibiting cells, but they were negative for γSMA. The figure was acquired employing the Leica Thunder system. Nuclei counterstained by DAPI. The bar is 100 μm

**Fig. 7:**
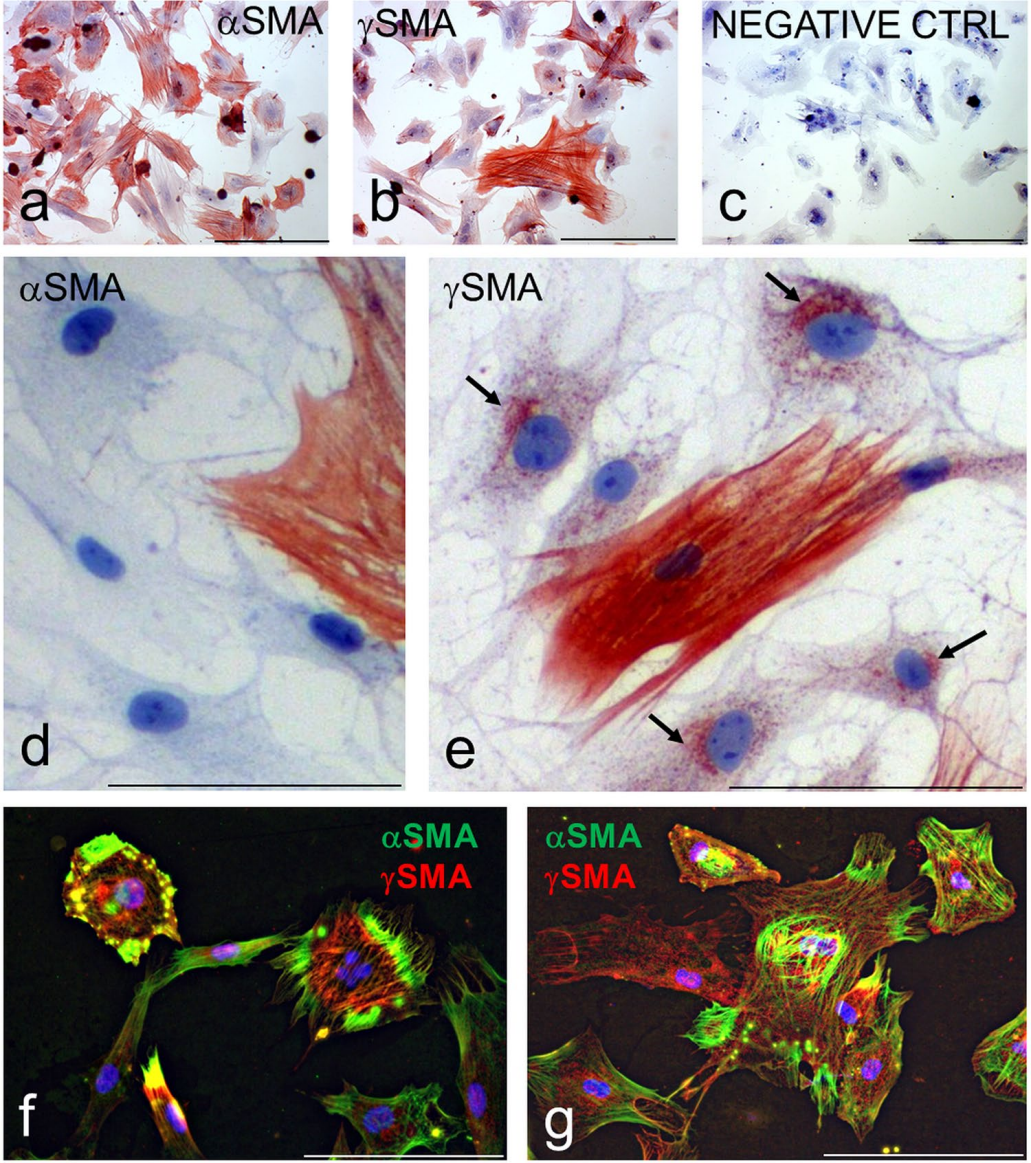
Detection of αSMA and γSMA in fibroblasts prepared from melanoma (MELF). MELF exhibited both αSMA (**a**) and γSMA (**b**) in comparison with the negative control (**c**). Cells without fibres of αSMA/γSMA exhibited no cytoplasmic signal of αSMA (**d**), in contrast to granules positive in γSMA with accumulation in the Golgi apparatus (arrows) (**e**). αSMA and γSMA (*f,g*) were co-expressed, but in different parts of the cell. Nuclei counterstained by haematoxylin (**a–e**) or DAPI (**f,g**). The bar is 100 μm

Immunofluorescence staining demonstrated that normal dermal fibroblasts were rarely positive for αSMA and negative for γSMA (Fig. 6). CAFs isolated from melanoma (MELF) with low activity of *ACTG2* exhibited distinct signals for both types of smooth muscle actins αSMA and γSMA under in vitro conditions (Fig. 7).

Numerous other CAFs exhibited a granular pattern of γSMA with accumulation of the signal in the Golgi apparatus (Fig. 7). A double labelling procedure at the single-cell level revealed that both actin proteins were occasionally present in one cell, while they occupied distinct compartments in the cytoplasm (Fig. 7).

**Fig. 8:**
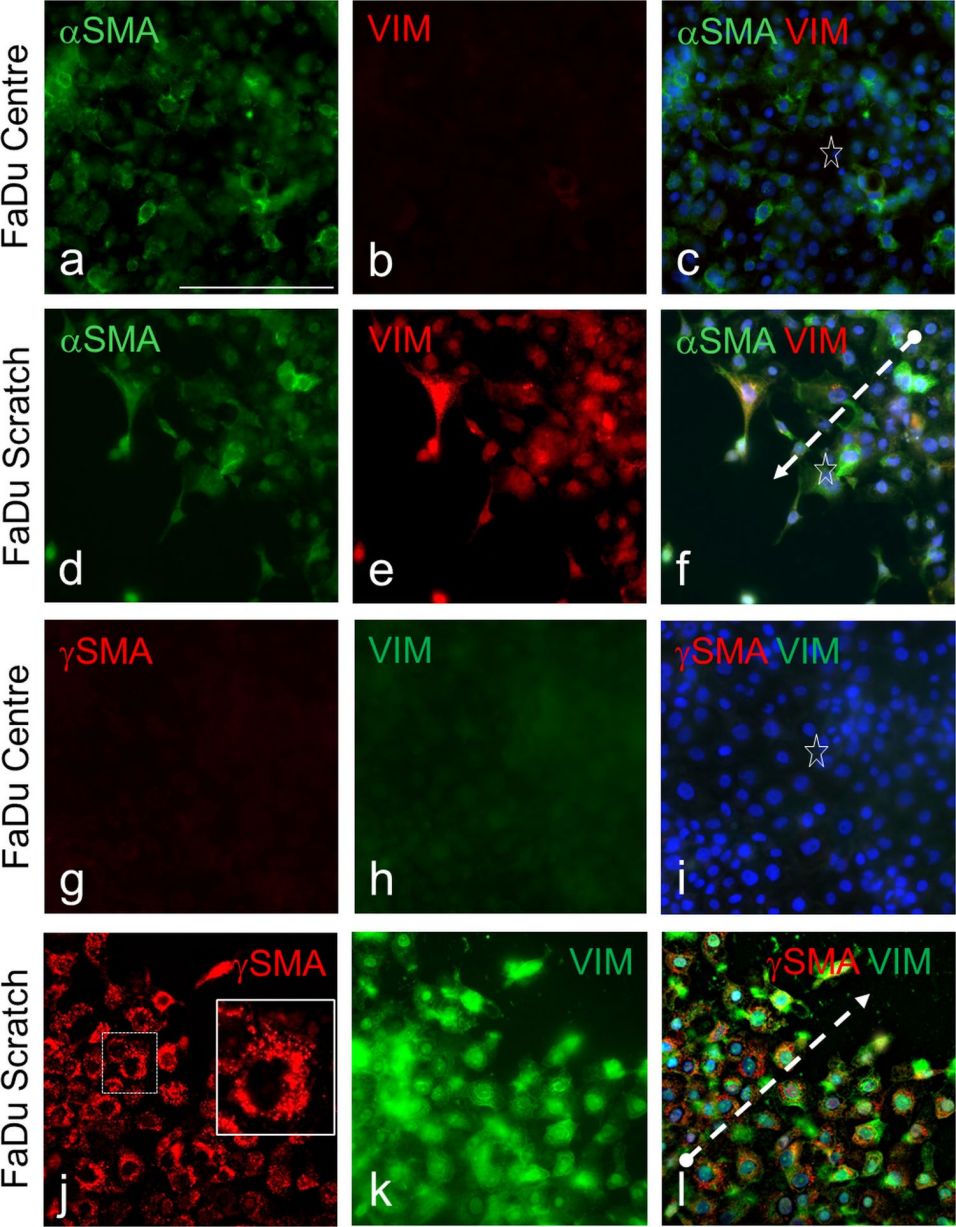
Comparison of the expression of αSMA or γSMA with vimentin (Vim) in in vitro culture of FaDu cells treated by the scratch test. The non-wounded centre of colonies is shown in panels *a–c* and *g–i*, and the margin of the wound is shown in panels **d–f** and **j–l**. Arrows are oriented in the direction of migration of FaDu cells from the periphery to the centre of the wound. The co-localization of both types of SMA in migrating cells is visible. Panel **j–l** (employing the Leica Thunder system) demonstrates the granular character of γSMA. Nuclei counterstained by DAPI. The bar is 100 μm

Because the possible involvement of γSMA was hypothesized in the context of epithelial-mesenchymal transition (Benzoubir et al. 2015), we tested the expression of both α/γSMA in cells of the FaDu line from hypopharyngeal cancer wounded by the scratch test, where we compared the expression of both proteins with mesenchymal marker vimentin (Vim). While the centre of colonies demonstrated only a low presence of αSMA-positive cells and an absence of cells positive for Vim and γSMA, both proteins, i.e., Vim and γSMA, were positive in cells migrating from the periphery of the scratch wound in the course of in vitro healing (Fig. 8). The positivity for γSMA in migrating FaDu was usually granular (Fig. 8). Cells positive for α/γSMA with fibroblast-like morphology were usually without contacts with other cells (Suppl. Figure 4). The studied fibroblasts isolated from human tissue expressed active genes producing mRNA for *SNAI2* (protein Slug), *FOXC2*, *CDH2* (N-cadherin), and *PCDH7* (Supplementary Figure 6). The expression of the *CDH1* gene (E-cadherin) was significantly lower in comparison with the activity of *CDH2* (N-cadherin) (Supplementary Figure 5).

**Fig. 9:**
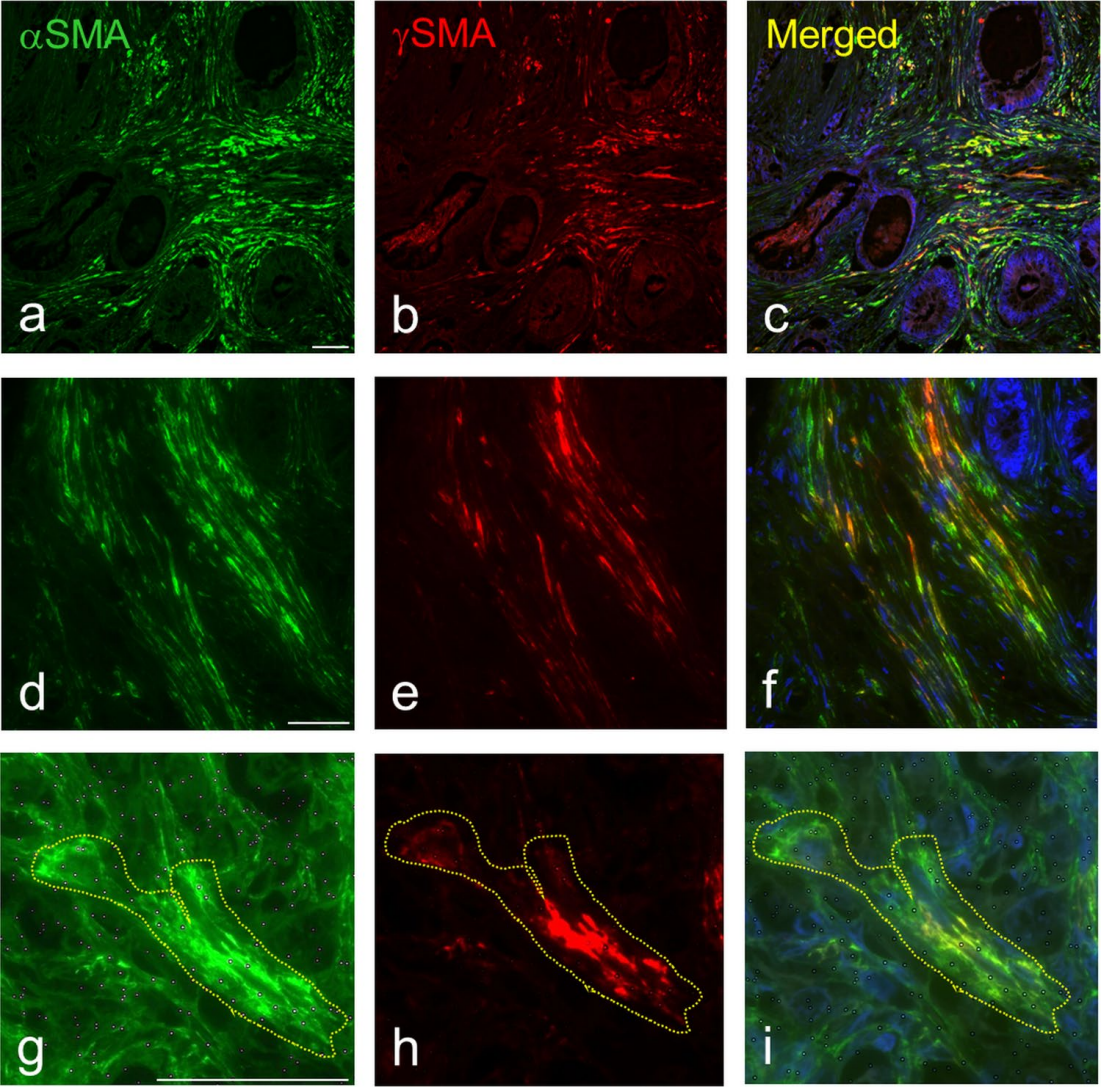
Metastasis of adenocarcinoma of the appendix to the ovary after simultaneous detection of αSMA and γSMA. (**a–i**) Co-expression of both types of SMA in CAFs is visible. The number of CAFs positive for αSMA is higher than that of double-positive cells (**a–f**). Double labelling also demonstrated that both types of SMA occupy different parts of the cell cytoplasm (**g–i**). Nuclei counterstained by DAPI. The bar is 100 μm

Double labelling demonstrated that the number of γSMA-positive CAFs was usually lower than the number of αSMA-positive CAFs (Fig. 9). We also observed tumours with αSMA-positive CAFs without γSMA-positive cells and samples negative for both types of SMA (Supplementary Figure 6). Rarely (one sample of aggressive carcinoma of the tongue), γSMA-positive CAFs predominated, with a negligible presence of αSMA-positive cells (Supplementary Figure 7). Double-labelled samples from clinical material (Fig. 9) also supported the results of in vitro observation, where both types of SMA were co-expressed at the single-cell level, but with different position in the cytoplasm.

## Discussion

Using immunohistochemistry, we have initially observed the presence of αSMA- and γSMA-positive stromal cells in tissue sections of various human malignant tumours.

It is well known that actins are evolutionarily one of the most conserved protein families in eukaryotes. This can be a significant disadvantage for immunohistochemical detection. Humans, like the majority of mammals, possess six different actin genes responsible for six different cell-type-specific isoforms. Due to the well-established high-sequence homology of these actin isoforms (Suresh and Diaz 2021), we intentionally selected antibodies targeting the more variable N-terminal domains of αSMA and γSMA.

Notably, our selected α-SMA-specific antibody (clone 1A4) was validated for its target specificity using an ACTA2 knockout HeLa cell line (edited by CRISPR/Cas9 technology). Using a publicly available database, we verified that the HeLa cell line expresses both ACTA2 and ACTG2 genes (https://www.proteinatlas.org/ENSG00000107796-ACTA2/cell+line; accessed 22 July 2025). Similar evidence can be presented for the FaDu cell line used in our experiments. This knockout model thus supports the strict specificity of the α-SMA antibody (clone 1A4).

It was observed by others (Shahid et al. 2018) at the protein level that, in caffeine-stimulated cells, αSMA and γSMA follow opposite trends. For western blotting quantification, they used our selected γSMA-specific antibody. This also suggests specificity of the γSMA antibody. While testing the specificity of the γSMA antibody in tissue sections, we also observed in consecutive tissue sections some γSMA positivity in structures lacking αSMA positivity (namely, in striated muscle). Notably, this confirms the diversity of antibody targets. This finding is also supported by independent evidence available in public databases (https://www.proteinatlas.org/ENSG00000163017-ACTG2/tissue/skeletal+muscle, accessed 8 August 2024). Thus, our data suggest, at the single-cell level, that the antibody used (ab189385) does not fully co-localize with 1A4.

In tissue sections, we frequently observed partial overlap of both actin isoforms. It was already known that in the murine model, smooth muscle γ-actin gene (Actg2) is expressed abundantly in visceral smooth muscle cells and with lower levels also in vascular smooth muscle cells (McHugh et al. 1991).

In detail, the issue of vascular smooth muscle γ-SMA was analysed by Sun and co-workers earlier (Sun et al. 2009). Similarly to our findings, using an ACTG2-selective antibody on sections of rat heart and aorta, these authors observed that levels of ACTG2 protein are present in the coronary artery, aorta, and some microvasculature, but not in cardiomyocytes or perivascular tissue. Similar spatial patterning of ACTA2/ACTG2 in vessels was observed by others as well. Arnoldi and co-workers suggested distinct functions of ACTG2- and ACTA2 in vascular smooth muscle cells (Arnoldi et al. 2013). Based on the spatial distribution of αSMA and γSMA, it is likely that ACTG2 primarily functions in the highly dynamic submembranous actin network, rather than in contractile filaments. Indeed, vascular smooth muscle cell contraction was not impaired by ACTG2 knockdown, but was reduced by ACTA2 knockdown.

Actin cytoskeleton polymerization dynamics near the membrane are also critical for the development of tension and transmission of generated force from the contractile apparatus to the extracellular matrix and to neighbouring cells. For these reasons, ACTG2 may have roles in cell migration, spreading, and resistance to passive stretch, with less critical roles in active contraction (Cipolla et al. 2002; Gunst and Zhang 2008; Hashmi et al. 2020). This can explain why αSMA and γSMA are sometimes, but not always, co-expressed in vessels (as in Supplementary Fig. 2).

In vitro, we tested these antibodies in various CAFs isolated from human tumour samples. We have observed αSMA-positive myCAFs in these cultures. Some of these myCAFs exhibited both αSMA and γSMA. Interestingly, γSMA was mostly co-expressed with αSMA in myCAFs, but the αSMA-positive cells without γSMA were more frequent than double-positive cells.

All studied cells isolated from the samples of normal human dermis (DF), distinct types of cancer tissue (CAFs), and tissues undergoing chronic inflammation (pancreatic PANF-control and SSF from systemic scleroderma) fulfilled the criteria required for fibroblast identification. This is evidenced by the activity of genes typical of this cell type (Muhl et al. 2020). However, we also observed vast differences among these various fibroblasts, as assessed by the number of differentially expressed genes. The relatively small difference between DF and CAFs was observed in the MELF (cutaneous melanoma, Dvořánková et al. 2017) and SSF (autoimmune scleroderma) samples. This can be explained by the recruitment of local fibroblasts in these conditions. Therefore, fibroblasts originating in the dermis are similar to DF. The vast difference between DF and normal fibroblasts from the pancreas suggests that a persistent transcriptional program underlies splanchnic lineage differentiation (Han et al. 2023a, b). Furthermore, these normal pancreatic fibroblasts acquired outside of the tumour niche could be affected by cancer indirectly. This can be explained by the systemic effect of cancer cells on the fibroblasts outside the tumour. We can speculate, for example, about the epigenetic nature of these modifications induced by systemic inflammatory reactions, necessary for, e.g., premetastatic niche formation (Plzák et al. 2010).

In 2020, Hashmi and co-workers suggested that in visceral myopathy, ACTG2 plays roles in functions that may depend on the submembranous actin network, namely, cell spreading and cell migration (Hashmi et al. 2020). This could be an immensely important topic in cancer biology. It is known that a mechanically active heterotypic E-cadherin/N-cadherin adhesion enables CAFs to drive cancer cell invasion (Labernadie et al. 2017). However, it is challenging to identify which specific subpopulation of CAFs is responsible for this invasion-promoting activity. Thus, we have focused on potential differences in αSMA and γSMA-positive myCAFs, respectively.

Our bioinformatics analysis indeed demonstrated that fibroblasts prepared from the pathological tissue differ from normal DF in pathways associated with development/morphogenesis, cell motility, and extracellular matrix production (Table 2). This interpretation fits well with the functional properties of CAFs described by others (Qin et al. 2024). Moreover, it agrees with KEGG (Kyoto Encyclopedia of Genes and Genomes) analysis demonstrating a significant difference from DF in “pathways in cancer” (accession hsa05200), where MELF, oMELF, PANF, PANF_c, SCCF, and SSF differ significantly.

All fibroblasts from pathological tissues demonstrate high expression of the *TPD52L1* gene. This was confirmed to be significantly different from normal DF. This gene is highly upregulated in cancer cells of malignant tumours such as breast (Boutros and Byrne 2005) and colorectal (Hong et al. 2021) cancer. However, the activation of this gene is also associated with the senescent status of dermal fibroblasts (Yoon et al. 2004). Functionally, CAFs are known as potent producers of various pro-inflammatory cytokines (Vokurka et al. 2022; D’Ambrosio and Gil 2023). Their tumour-supporting secretome significantly overlaps with the senescence-associated secretory phenotype (Takasugi et al. 2022). This can be illustrated by the active gene for IL-6 detected in cells in our study. Secretion of IL-6 is one of the characteristic features of activated fibroblasts, including CAFs (Španko et al. 2021; Xiang et al. 2022). The studied cells also exhibited high activity of gene *IL6ST* as a component of the IL-6 receptor complex. However, only negligible activity of the gene encoding the IL-6 receptor was observed across the fibroblasts (Table 3).

**Table 3:** Differences between normal dermal fibroblasts and fibroblasts from pathological tissue

Accession	Term	*p*-value
GO:0032502	Developmental process	2.19e−18
GO:0048731	System development	4.37e−18
GO:0009653	Anatomical structure morphogenesis	4.65e−17
GO:0016477	Cell migration	1.90e−13
GO:0040011	Locomotion	5.23e−13
GO:0048870	Cell motility	6.99e−13
GO:0005578	Proteinaceous extracellular matrix	4.80e−10
GO:0031012	Extracellular matrix	6.26e−10
GO:0044420	Extracellular matrix component	3.87e−06

This seemingly paradoxical finding can be explained by mechanistic differences in IL-6 classical and IL-6 trans-signalling. It has recently been well established that the pro-inflammatory roles of IL-6 are due to the trans-signalling pathway, whereas anti-inflammatory and regenerative signalling is mediated by IL-6 classic signalling (Wolf et al. 2014). Thus, CAFs lacking IL-6R are not responsive to anti-inflammatory IL-6 classic signalling. Conversely, expressed gp130 allows these CAFs to act in an inflammation-maintaining and thus pro-tumorigenic manner as a result of IL-6 trans-signalling.

Contrary to the high activity of *TPD52L1* in fibroblasts from pathological tissue, these cells exhibited only negligible activity of gene *CLEC12A* compared to the normal DF. This gene encodes a lectin expressed predominantly by granulocytes (McLeish and Fernandes 2023). Recognition of uric acid crystals by this lectin reduces activation of immune cells by dead cells (Neumann et al. 2014). On the other hand, this lectin also positively influences the delivery of antigens to the myeloid antigen-presenting cells (Jung et al. 2016). The absence of an active gene in CAFs and pathologically activated fibroblasts, including PAN_control and SSF, can influence the immune microenvironment in the tissue to acquire inflammation-supporting properties typical of a tumour niche.

Activation of gene *MFAP5* across all CAFs (except oMELF) is typical of fibroblasts present in malignant tumours (Peng et al. 2023), where these cells promote malignant cells to form metastases (Wang et al. 2023). This protein, expressed by fibroblasts, also positively correlates with scarring. This *MFAP5* gene activity is also detected in SSF, as fibroplasia is a hallmark of scleroderma (Han et al. 2023a). The MFAP5 protein influences properties of the extracellular matrix in cancer, including collagen deposition, and its targeting should be suitable for adjuvant cancer therapy (Duan et al. 2023).

CAFs (except MELF), PANF_c, and SSF exhibited high activity of gene *ACTG2* encoding γSMA. A similar pattern was observed for the expression of genes *ABLIM1*, *CNN1,* and *CALD1.* Their products seem to be related to the function of actin, as was observed in these cells (He et al. 2024; Huang et al. 2024; Wu et al. 2024). The typical CAFs contain fibres of polymeric γSMA in the cytoplasm that are located differently from αSMA. Moreover, some CAFs exhibited a granular pattern of γSMA, with accumulation in the area of the Golgi apparatus.

Experiments performed with malignant epithelial cells from the human hypopharynx (FaDu) demonstrated that γSMA is expressed in cells positive for Vim with fibroblast-like morphology. These cells were usually devoid of contact with other cells. They also exhibited αSMA. The transcriptome of the studied fibroblasts exhibited active gene *SNAI2* (encoding protein slug), *FOXC2*, *CDH2* (N-cadherin), and *PCDH7*, and low expression of *CDH1* (E-cadherin). The role of these genes in epithelial-mesenchymal transition has been described (Shimoda et al. 2018; Nam et al. 2022; Khan et al. 2024). The role of γSMA in cancer in the epithelial-mesenchymal transition was described in hepatocellular carcinoma and cancer of the prostate and oesophagus (Lin and Chuang 2012; Benzoubir et al. 2015; Chen et al. 2020; Lei et al. 2022). Cells with γSMA are important in the process of formation of keloid scars (Qiu et al. 2024). On the other hand, under physiological conditions, these cells participate in the skin appendage development in the rat (Morioka and Takano-Ohmuro 2016). The participation of keratins such as keratin 8 or 18 (Dvoránková et al. 2005; Jung et al. 2016; Ceausu et al. 2018) is an important clue for the epithelial-mesenchymal transition. Indeed, we observed the active *KRT18* gene in the studied fibroblasts. Our observation correlates with published data and suggests a possible role of epithelial-mesenchymal transition in the formation of γSMA-positive fibroblasts. The epithelial-mesenchymal transition is, besides its role in cancer, also commonly considered as a mechanism in systemic sclerosis (Nakamura and Tokura 2011; Zhang et al. 2022) and pulmonary and renal fibrosis (Chapman 2011; Allison 2015). CAFs are frequently formed by the transition of fibroblasts to myofibroblasts under the influence of TGF-β1. This process positively influences the activation of the *ACTG2* gene (Untergasser et al. 2005).

Genetic analysis demonstrated that the activity of the *ACTG2* gene is positively associated with the malignant transformation of epithelial cells (Jin et al. 2023; Yang et al. 2024). This gene also influences the growth and metastatic spread of prostate cancer (Wu et al. 2017) (Wu et al. 2017; Huang et al. 2024 b). However, in distinctive types of cancer, such as bladder cancer, it has anti-proliferative and pro-apoptotic effects (Liu et al. 2023). These data show that the activity of the *ACTG2* gene is multifaceted, and it can have some clinical relevance and warrants further research.

In conclusion, γSMA is usually co-expressed with αSMA in part of myCAFs. Their origin is not clear, but the observation of Benzoubir and co-workers (2015) and the data presented in this study permit the hypothesis that epithelial-to-mesenchymal transition can have some role in the origin of part of γSMA-positive CAFs; however, this needs to be verified experimentally in the future. The potential relation between the presence of γSMA-positive fibroblasts in the cancer microenvironment and the biological properties of tumours needs to be studied.

## Supplementary Information

Below is the link to the electronic supplementary material.

Supplementary file1 (DOCX 19834 KB)

## Data Availability

The MIAME-compliant data were deposited to the ArrayExpress database under accession numbers E-MTAB-8764 and E-MTAB-15008.
