# Supplementary material for: Gamma smooth muscle actin as a new potential marker of cancer-associated fibroblasts
Source: Histochem Cell Biol. 2025 Sep 19;163(1):93. doi: 10.1007/s00418-025-02419-9 (PMC12449396; doi:10.1007/s00418-025-02419-9)
Supplement: Supplementary file 1 — Supplementary file1 (DOCX 19834 KB) [file 418_2025_2419_MOESM1_ESM.docx]

**Gamma smooth muscle actin as a new potential marker of cancer-associated fibroblasts**

*Histochemistry and Cell Biology*

Michal Španko^1,2,5,x^, Lucie Pfeiferová^3,x^, Eliška Drobná Krejčí^1^, Michal Kolář^3^, Pavel Dundr^4,5^, Jaroslav Valach^2,5^, Karel Smetana, Jr.^1^, Lukáš Lacina^1,5*^

**Supplementary figures**

**
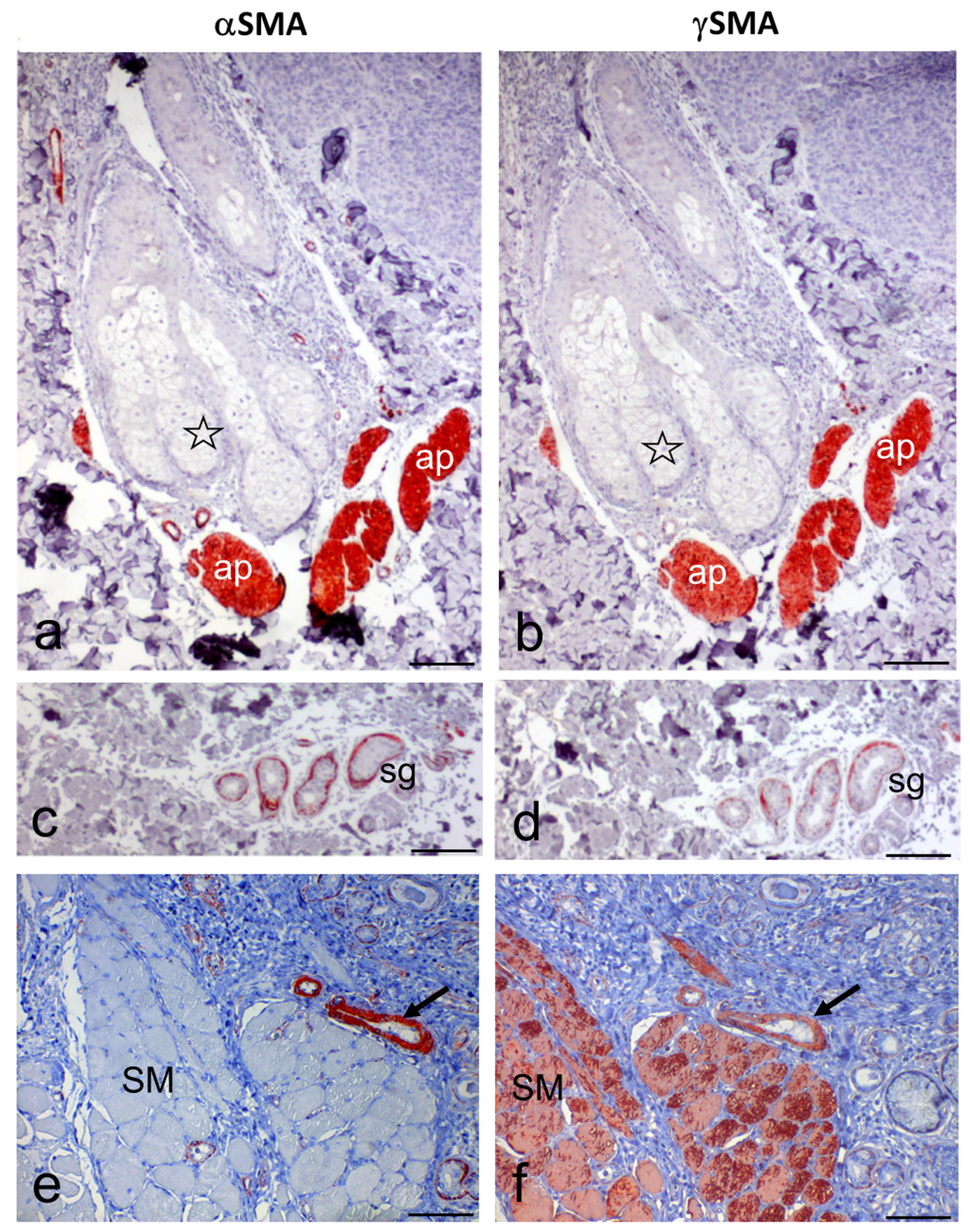
**

**Suppl. Fig. 1. Detection of αSMA (a,c,e) and γSMA (b,d,f) in sections from normal skin and muscle.** The sebaceous gland (asterisk) is negative in contrast to the highly positive arrector pilli muscle (ap). Myoepithelial cells of sweat glands (sg) are positive, as well as smooth muscle cells of vessels (arrows). Striated muscle fibres are positive for γSMA. Counterstained by Gill´s haematoxylin. The bar is 100 μm.

**
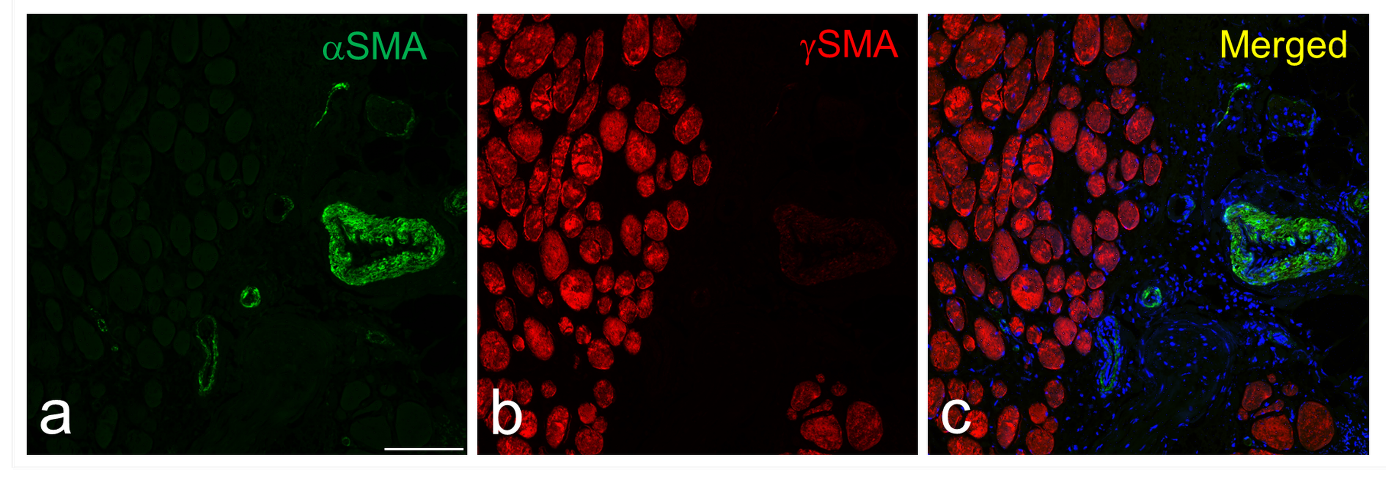
**

**Suppl. Fig. 2. Detection of αSMA (a) and γSMA (b) in sections from the tongue.** The smooth muscle cells in vessels express αSMA and muscle fibres are positive for γSMA. Nuclei were counterstained by DAPI (c). The bar is 100 μm.

**
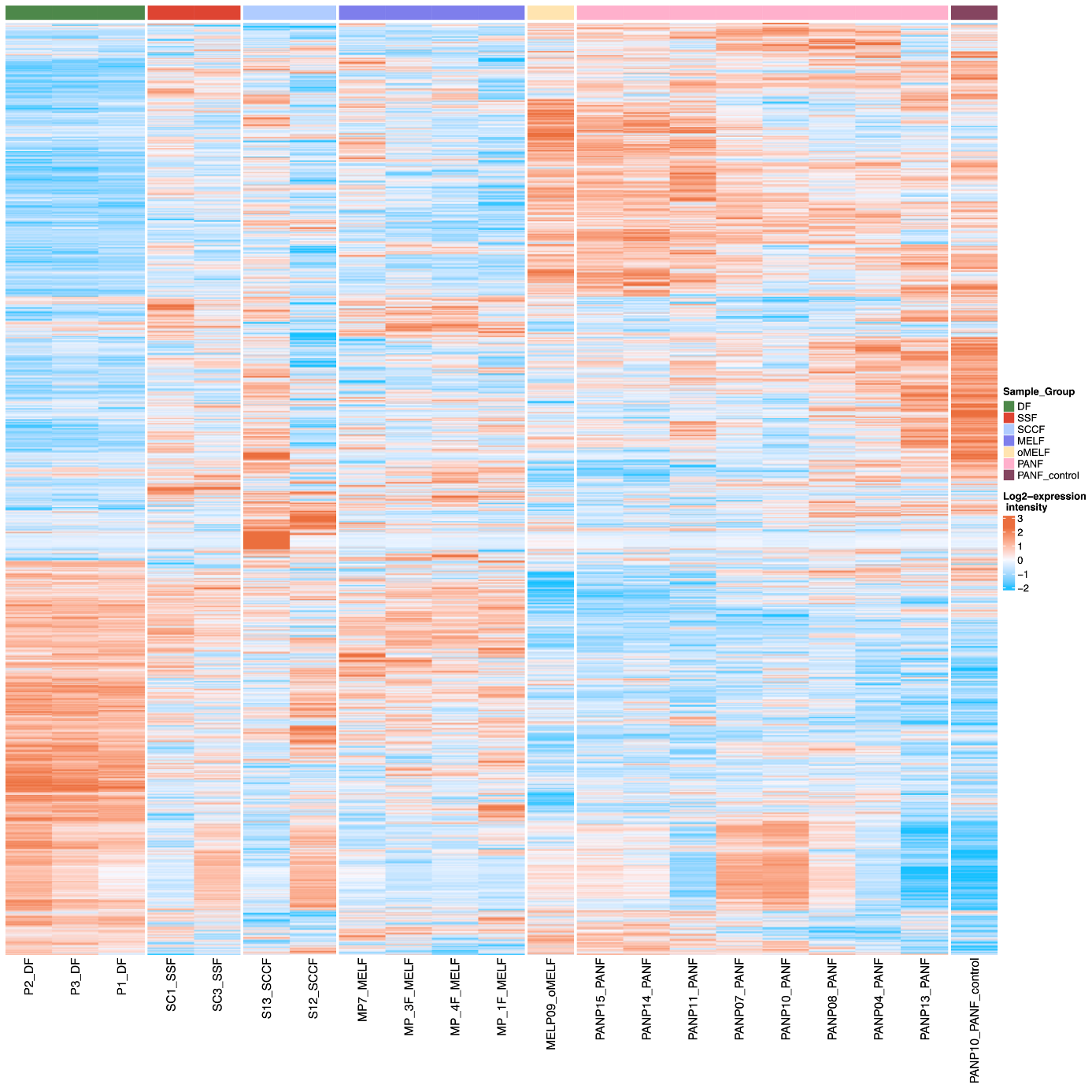
**

**Suppl. Fig. 3. Heatmap demonstrating that normal dermal fibroblasts (DF) differ from CAFs (MELF, PANF, SCCF, oMELF), normal fibroblasts from cancer-affected pancreas (PANF_C), and fibroblasts from systemic sclerosis (SSF)**


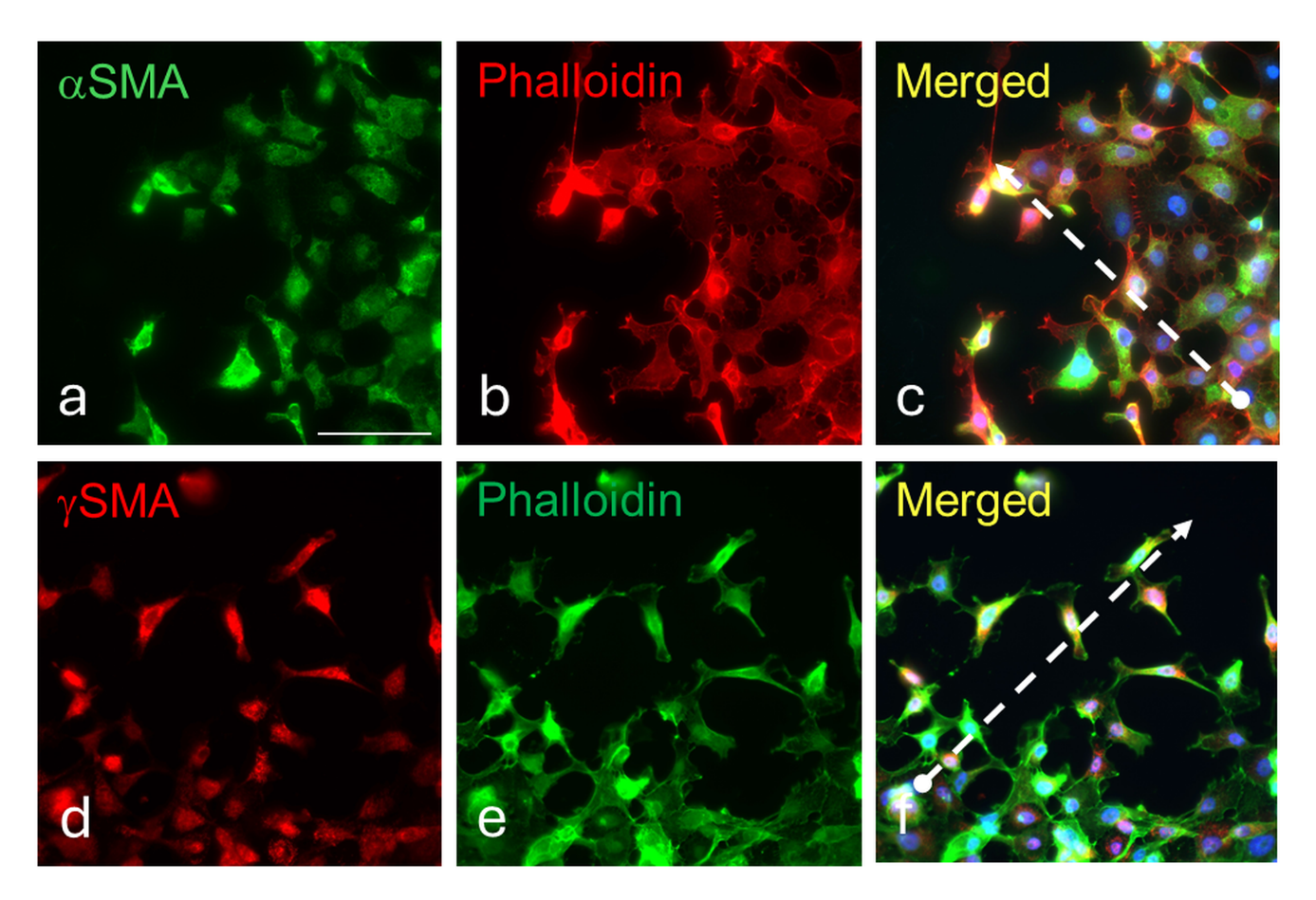


**Suppl. Fig. 4. FaDu cells with mesenchymal morphology migrating from the periphery to the wound centre exhibiting αSMA (a) and γSMA (d).** The direction of fibroblast-like cell migration is marked by the arrow. F-actin was labelled by phalloidin (b,e). Nuclei were counterstained by DAPI (c,f). The bar is 100 μm.


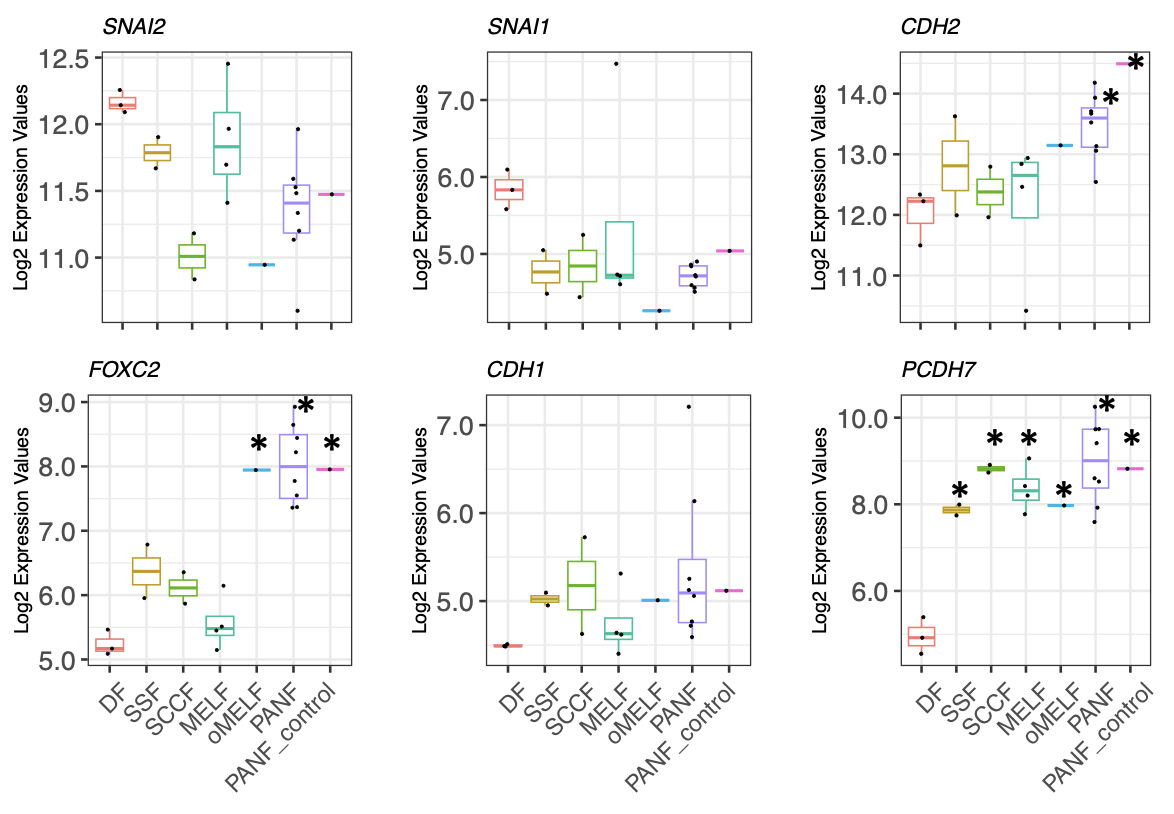


**Suppl. Fig. 5. Detection of selected genes in studied fibroblasts.** While all fibroblasts exhibited low activity of gene *SNAI1* (snail), the *SNAI2* (slug) gene was highly expressed, as well as the gene encoding *FOXC2*, whose activity was upregulated in PANF, PANF_C, and oMELF. The activity of *CDH1* (E-cadherin) was negligible, which contrasts with the high activity of *CDH2* (N-cadherin). The activity of the *PCDH7* gene was significantly higher (p<0.05) in CAFS, PANF_c, and SSF than in normal DF. These data indicate that the relation to epithelial-mesenchymal transition seems to be probable.

**
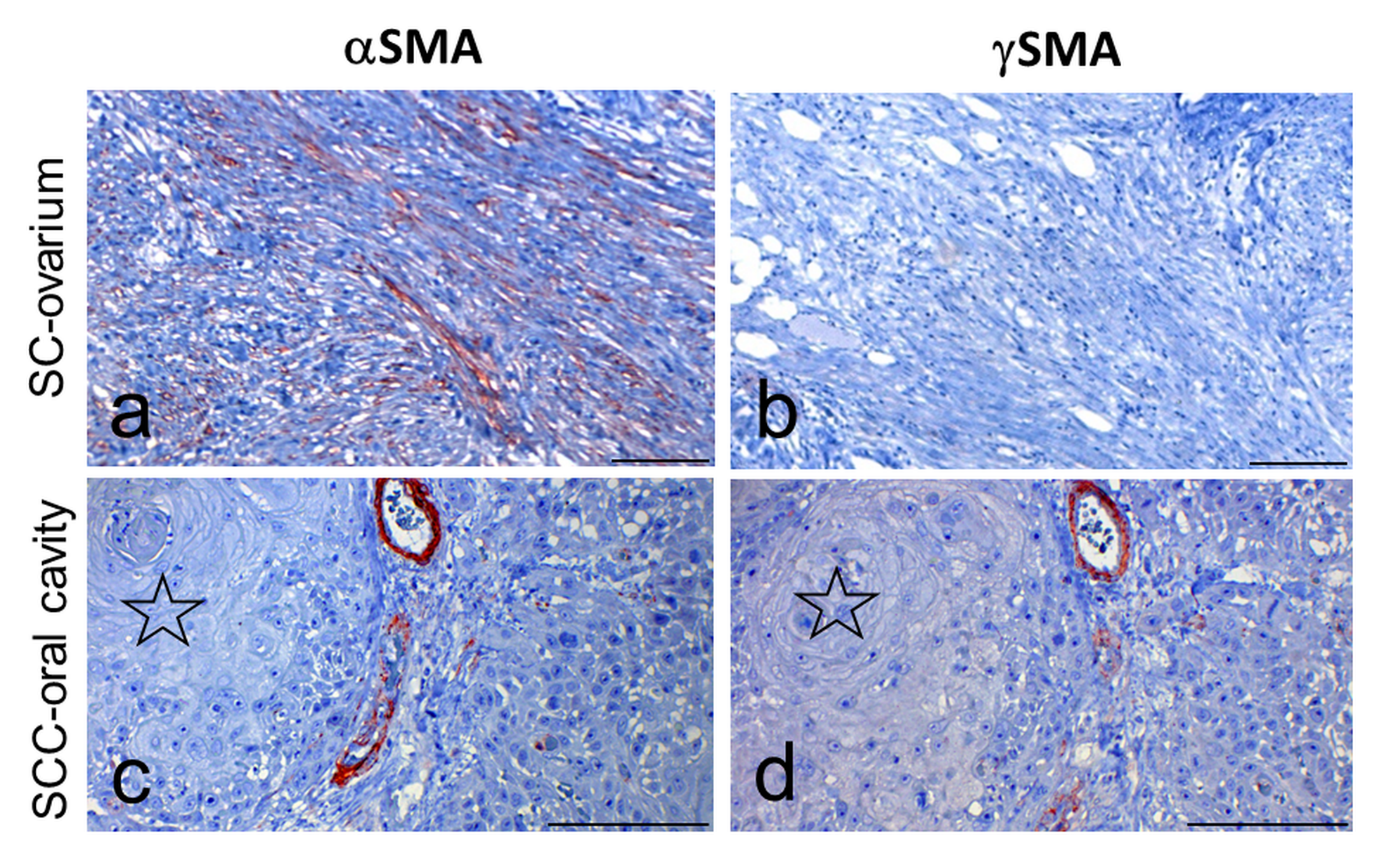
**

**Suppl. Fig. 6.** Tumours with αSMA-positive CAFs can also be without γSMA-positive cells, as observed in a sample of high-grade serous carcinoma of the ovary (a,b). Samples without both types of actin were also noted, similarly as in the example of squamous cell carcinoma of the oral cavity (c,d). Positive smooth muscle cells of vascular wall can be used as an internal positive control. Keratin pearls are marked by an asterisk. Counterstained by Gill´s haematoxylin. The bar is 100 μm.


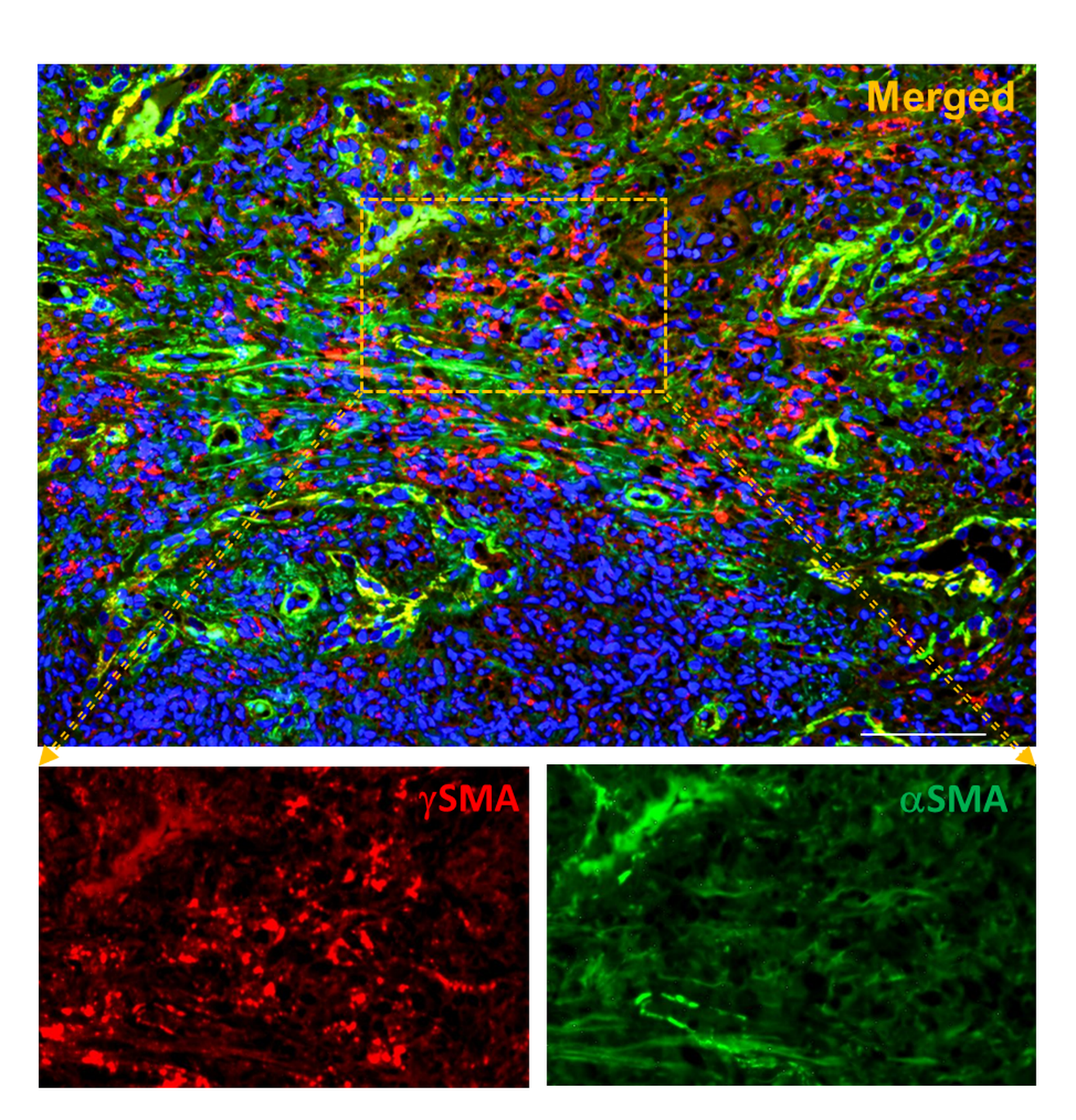


**Suppl. Fig. 7. Detection of αSMA and γSMA in the section from aggressive squamous cell cancer of the tongue.** αSMA positivity is present in the smooth muscle cells of the vascular wall. Numerous γSMA-positive cells are located in the tumour stroma. Nuclei counterstained by DAPI. The bar is 100 μm.
